# Molecular forms of the Indian *Anopheles subpictus* complex differ in their susceptibility to insecticides and the presence of knockdown resistance (*kdr*) mutations in the voltage-gated sodium channel

**DOI:** 10.1371/journal.pone.0280289

**Published:** 2023-02-02

**Authors:** Ankita Sindhania, Himanshu P. Lohani, Madhavinadha Prasad Kona, Taranjeet Kaur, B. R. Kaushal, Om P. Singh

**Affiliations:** 1 National Institute of Malaria Research, Dwarka, New Delhi, India; 2 Department of Zoology, Kumaun University, Nainital, India; Institute of Zoology Chinese Academy of Sciences, CHINA

## Abstract

**Objectives:**

To investigate the differential insecticide-susceptibility of two molecular forms of *Anopheles subpictus* complex (A and B) against DDT and pyrethroids, the occurrence of knockdown resistance (*kdr*) mutations in these forms, and the association of *kdr* mutations with insecticide resistance.

**Methods:**

Insecticide susceptibility tests of *An*. *subpictus s*.*l*., collected from coastal and inland areas of mainland India, were performed against DDT, permethrin and deltamethrin using the WHO standard insecticide susceptibility test kit. The mosquitoes were characterized for molecular forms using a diagnostic PCR developed in this study. Representative samples of *An*. *subpictus* molecular forms A and B were sequenced for a genomic region encompassing the IIS4-5 linker to the IIS6 segments of the voltage-gated sodium channel to identify *kdr* mutations. A common PIRA-PCR was developed for identifying L1014F-*kdr* mutation and used for genotyping in both molecular forms of *An*. *subpictus*.

**Results:**

Molecular form A of *An*. *subpictus* was resistant to all three insecticides, i.e., DDT, Permethrin and deltamethrin, whereas Form B was categorized as ‘possibly resistant’ to these insecticides. Significantly higher mortalities in WHO insecticide susceptibility tests were recorded in Form B compared to Form A in sympatric populations. Molecular characterization of the IIS4-5 linker to IIS-6 segments of the voltage-gated sodium channel revealed the presence of two alternative nucleotide transversions at L1014 residue in Form A, both leading to the same amino acid change, i.e., Leu-to-Phe; however, such mutations could not be observed in Form B. PIRA-PCR-based *kdr-*genotyping of field populations revealed high frequencies of L1014F-*kdr* mutations in Form A and the absence of this mutation in Form B. The proportion of L1014F mutation was significantly higher in resistant mosquitoes following insecticide-bioassay with DDT (*p*<0.0001), permethrin (*p*<0.001) and deltamethrin (*p*<0.01) as compared to their susceptible counterparts.

**Conclusions:**

Significant differences in insecticide susceptibility were found between two molecular forms of *An*. *subpictus* complex in sympatric populations. The L1014F-*kdr* mutation was observed in Form A only, which was found to be associated with DDT, permethrin and deltamethrin resistance.

## Introduction

*Anopheles subpictus s*.*l*. is widely distributed in the Oriental Region and some parts of the Australasian region [1–3]. It has been recognized as a primary malaria vector in Australasian Zone, Celebes, Portuguese Timor and South East Asia and as a secondary vector in Sri Lanka [2–4]. In India, this species is not listed as a malaria vector [5], but reports suggest that this species is an important malaria vector, at least in coastal areas [6–7], where species B of the *An*. *subpictus* dominates [8–11]. The variation in the vectorial role may be due to the presence of different biological species. The taxonomy of *An*. *subpictus s*.*l*. is highly complex. In India, Suguna et al. [9] identified four sibling species, provisionally designated as species A, B, C and D, based on two inversions located on chromosome X (Xa and Xb). Suguna et al. [9] also established that these sibling species can be identified based on the mode number of ridges present on the egg float. Further molecular characterization of *An*. *subpictus* from the Indian subcontinent revealed the presence of three molecular forms, namely Form A, B and C [10]. Form A was prevalent throughout mainland India and Sri Lanka. Form B was present in the coastal regions of India and Sri Lanka, and Form C was present in Andaman & Nicobar Islands. Correlation of molecular form with the number of ridges present on the egg float revealed that molecular form B corresponds to species B, but the majority (80%) of molecular form A corresponds to species C and the rest to species A and D. The chromosomal inversions characteristics of molecular forms have not been studied yet. Phylogenetic studies based on ITS2, 28S-rDNA and mtDNA revealed that Form B is closely related to members of the Sundaicus Complex and is far distantly related to Form A. Surendran et al. [12] and Sindhania et al. [10], therefore recommended classifying species B as molecular form B under the Sundaicus Complex. Moreover, Sivabalakrishnan et al. [13] have described species B as *An*. *sundaicus s*.*l*. Wilai et al. [14] have recently suggested the presence of two species within *An*. *subpictus* in Thailand, and more than one species in Indonesia based on ITS2 (ribosomal DNA) and cytochrome oxidase-1 (CO1, mitochondrial DNA) sequences but none of them is identical to any molecular forms present in the Indian subcontinent but is closely related to molecular form B.

Synthetic pyrethroids are the main insecticides being used in India for Indoor Residual Spraying (IRS) and for the Long Lasting Insecticidal Nets (LLINs) in addition to DDT and malathion. Carbamate has never been used in India. The insecticide susceptibility of *An*. *subpictus* is poorly studied in the Indian population [2] and there is no report on the differential susceptibility of different sibling species of *An*. *subpictus* in India. In this study, we report the differential susceptibility of two molecular forms prevalent in India (A and B) against DDT and pyrethroids, as well as the presence of knockdown resistance (*kdr*) mutations that are known to confer reduced sensitivity to these insecticides.

In this study, we classified and described the molecularly identified sibling species of *An*. *subpictus* as molecular forms A and B following Sindhania et al. [10] in absence of correlation of these molecular forms with cytologically identified sibling species (A, B, C and D).

## Material and methods

### Selection of the study areas and mosquito collection

For determining the differential susceptibility of molecular forms of *An*. *subpictus*, two coastal areas were selected where molecular forms A and B are present in sympatric association based on the previous study by Sindhania et al. [10]: (i) villages near Chilka lake which is a large brackish water lagoon covering an area of over 1100 km^2^ in Odisha state (eastern India), and (ii) villages near Puducherry (non-contiguous enclaves). The villages from where mosquitoes were collected near Chilka lake were Panasapada, Satapada, Brahmgiri, Sipakuda, Minsa, Siara, Gambhari (19° 18–73′, 85° 04–47′ E) and near Puducherry were Kaliankuppam, Munjalkuppam, Sedarapet and Pillayarkuppam (11° 48′-12° 05′ N, 79° 44′-76′ E). In addition, mosquitoes were also collected from inland areas in northern India (Nuh, Haryana; 27° 78’ N, 77° 23’) where molecular form A is allopatric. Female *An*. *subpictus s*.*l*. were collected from their resting habitats (human dwellings and cattle sheds) in the morning between 6:00 to 8:00 AM with the help of a mouth aspirator and flash torch and brought alive in a field laboratory, where mosquitoes were maintained in an insect cage with access to cotton pads soaked with 10% glucose and water. Once the mosquitoes had attained a gravid stage, individual mosquitoes were morphologically identified in a test tube under a stereo microscope. Morphologically identified *An*. *subpictus s*.*l*. were pooled in a mosquito cage to lay their eggs. F1 progeny was obtained from a pool of 25–150 mosquitoes from each collection. The progenies were reared till their emergence into adults following the method described by Sharma et al [15].

### Insecticide susceptibility assays

Insecticide susceptibility assays were performed using the WHO adult mosquito insecticide susceptibility test on three-to-four-days-old and sugar-fed adult female mosquitoes (F1). Up to 25 *An*. *subpictus s*.*l*. mosquitoes were transferred in each holding tube provided with the kit which were subsequently transferred to an exposure tube lined with the insecticide paper impregnated with diagnostic doses (4% DDT, 0.75% permethrin and 0.05% deltamethrin) along with appropriate controls. Mosquitoes were exposed to insecticide-impregnated paper for one hour and transferred back to the holding tubes and placed in a field laboratory maintained at a temperature of 25±1°C. A cotton pad soaked in sugar solution was placed on top of the tube during recovery to maintain adequate humidity. Dead and alive mosquitoes after 24 hours of insecticide exposure were re-identified for species under a dissecting microscope. The corrected mortalities of *An*. *subpictus s*.*l*. were estimated. The alive and dead mosquitoes tested against each insecticide were preserved in microfuge tubes containing isopropanol for molecular studies.

### DNA isolation

DNA was isolated from individual mosquitoes by the method of Black and Duteau [16]

### Species identification

**Fig 1:**
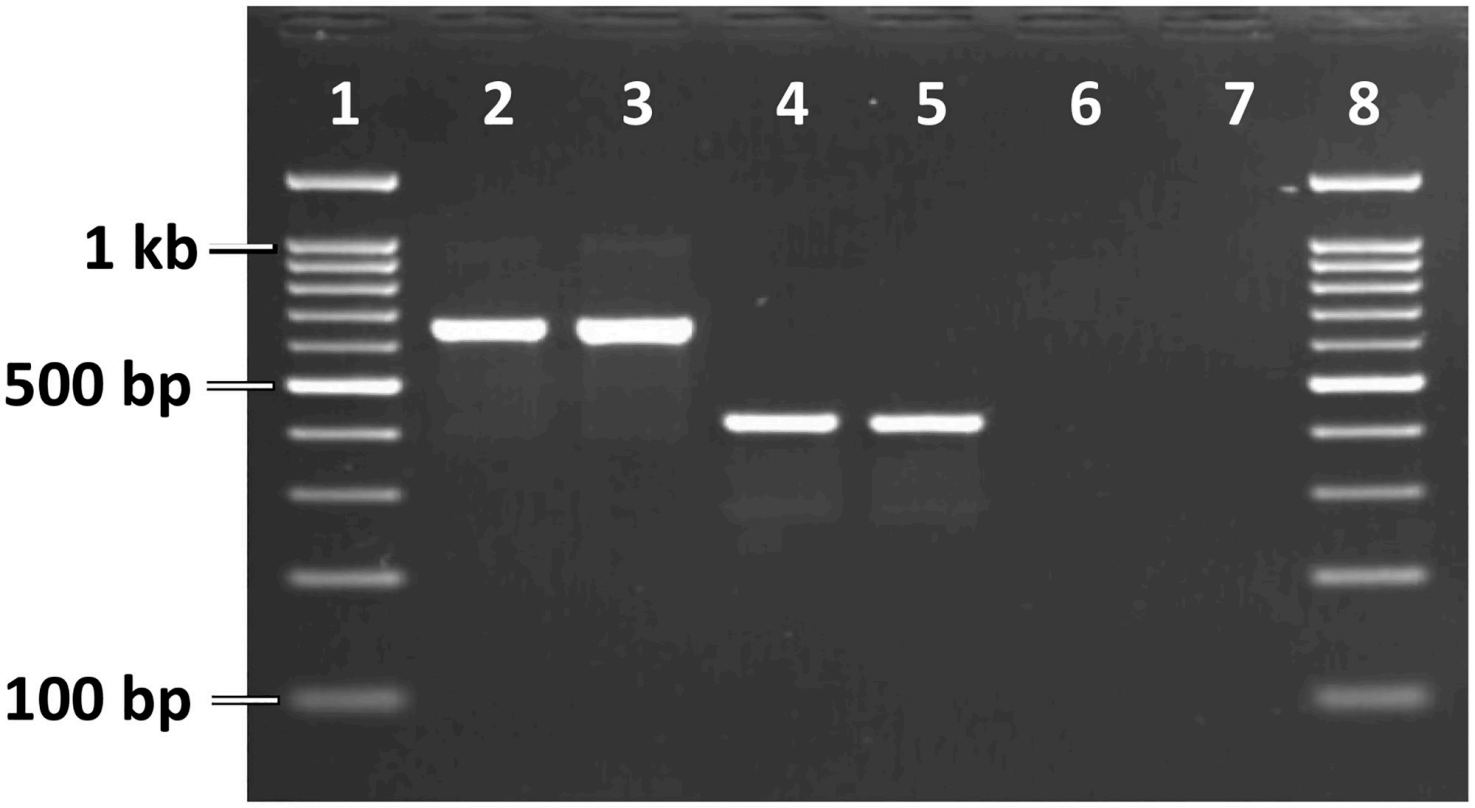
PCR assay for the identification of molecular forms of *An*. *subpictus*. Lanes 1 and 8: 100 bp ladder; lanes 2–3: Form A; Lanes 4–5: Form B; lanes 6: *An*. *vagus*; lane 7: negative control, without DNA.

Morphological identification of *An*. *subpictus s*.*l*. was performed following keys by Christophers [1]. For the identification of molecular forms of *An*. *subpictus*, a new PCR assay was developed due to the limitations of previously designed methods for this purpose. The earlier molecular methods developed by Surendran et al. [17] and Sindhania et al [10] had shown cross-reactivity with *An*. *stephensi* and *An*. *vagus*, respectively, both of which can be mistaken as *An*. *subpictus s*.*l*. morphologically in some cases where specimens have lost their scales. Two new species-specific primers, SubA1R and SubB1R, were designed which were specific to molecular form A and B, respectively and used with the universal primer SubF designed by Surendran et al. [17]. Similarity search (BLASTn) of species-specific primers confirmed that these primers do not have complementarity with ITS2 of any other mosquito species specifically at 3´ end. The nucleotide sequences of the primers have been shown in Table 1. The expected size of the diagnostic amplicon in Form A and Form B was 645 and 411 bp respectively. PCR amplification was carried out in a reaction mixture of 20 μl using GoTaq Green Master Mix 2X (Promega) with 0.25 μM each primer (SubF, SubA1R, and SubB1R). The PCR conditions were: initial denaturation at 95°C for 3 min followed by 35 cycles each with 95°C for 30 sec, 58°C for 30 sec, 72°C for 1 min and a final extension at 72°C for 7 min. Three μL of PCR product was electrophoresed on 2.0% agarose gel and visualized under UV (Fig 1). The PCR didn’t show cross-reactivity with DNA isolated from three non-target anophelines i.e., *An*. *stephensi*, *An*. *vagus* and *An*. *sundaicus* which may be misidentified as *An*. *subpictus* in case of lost hair and scales.

**Table 1:** List of primers used in this study.

Primers’ name	Nucleotide sequence (5´-3´)	Reference
**Primers for the identification of molecular forms**		
1. SubF	ACTGCAGGACACATGAACACCG	**Surendran et al. [17]**
2. SubA1R	GTGTTTGGCAACTCTCATC	**This study**
3. SubB1R	CGGTTGATACAGGACGCA	**This study**
**Primers for DNA sequencing of the voltage-gated sodium channel**		
1. Sub1F	GAGTGTTTAAGCTCGCCAAA	**This study**
2. Sub1R	TCTTTCCGAACAGCTGCATT	**This study**
3. Sub2F	GGCAAACGCGCAAGAAATTG	**This study**
4. Sub2R	GACAAAAGCAAAGCTAAGAAAAG	**This study**
**Primers for *kdr-*genotyping**		
1. Sub_kdF	gactgactgactgactgactTCTTAGCTACGGTAGTAATAGGAAA**A**TT *	**This study**
2. SubA_kdR	CACCTGCAAAACAATAACATGTT**C**AATTC *	**Singh et al. [19]**
3. SubB_kdR	AAACAGTGAGAAGTGAGCCG	**This study**

*The small typeface letters of the nucleotide sequence are primer tail and bold-underlined letters represent the intentional mismatch introduced in the primer.

### Molecular characterization of VGSC for the identification of *kdr* mutations

**Fig 2:**
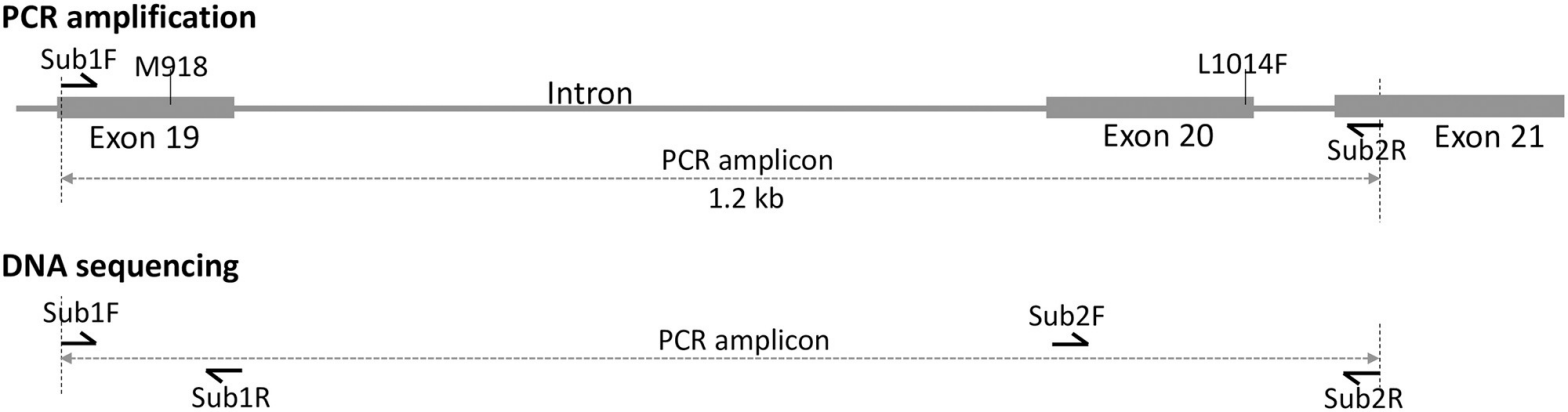
Schematic presentation of the location of primers (represented by harpoons) used for PCR amplification of partial VGSC (IIS4-S6) and sequencing. Exon numbering is based on the VectorBase (https://vectorbase.org) sequence of the voltage-gated sodium channel of *An*. *epiroticus* (Gene ID: AEPI015231; retrieved: 15 Sept 2022).

For the identification of *kdr* mutations in the VGSC of *An*. *subpictus*, primers were designed for the amplification of partial VGSC covering a region of IIS4-5 linker to IIS6 segments, which included two *kdr* locus M914 and L1014 residues (amino acid positions based on housefly sequence) based on sequences available for *An*. *epiroticus*, which is a closely related species of *An*. *subpictus* [10]. PCR products from representative samples of molecular forms A and B, which were resistant to DDT and pyrethroids were sequenced for partial VGSC. The PCR amplification and DNA sequencing strategies have been displayed in Fig 2. The PCR was amplified using primers Sub1F and Sub2R covering *kdr* loci M918T (super-*kdr*) and L1014F/C/S (*kdr*). PCR amplification was carried out in a reaction mixture (20 μl) using DreamTaq Green PCR Master Mix (2X) (ThermoFisher Scientific) with 0.5 μM of primer Sub1F, and Sub2R. The PCR conditions were: initial denaturation at 95°C for 5 min followed by 35 cycles each with 95°C for 30 sec, 52°C for 30 sec, 72°C for 90 sec and a final extension at 72°C for 7 min. 3 μL of the PCR product was electrophoresed on 1.5% agarose gel, visualized under UV, and the remaining PCR products were purified using Exo-Sap IT (Thermo Fisher Scientific) and subjected to sequence termination reaction using BigDye Terminator v3.1 Cycle Sequencing Kit (Applied Biosystem). Since initial sequencing using these two primers often failed due to indel present in the intron between two exons containing the above-mentioned *kdr* mutations, each PCR product was subjected to four sequencing termination reactions using two additional internal primers Sub1R and Sub2F (Table 1) in addition to primers used for PCR amplification. The product was purified and electrophoresed in ABI Prism 3730xl following the vendor’s protocol. The sequences were aligned using MUSCLE implemented in MEGA-7 [18]. The sequences have been submitted to GenBank (accession nos. OP846124-OP846347).

### Molecular assay for *kdr*-genotyping

Since there are two alternative mutations leading to the same amino acid substitution, and the downstream sequence immediately following L1014 residue (intron) is highly diverse among two molecular forms, this needs the development of multiple ASPCR (for two mutations and two molecular forms). We, therefore, designed a single PIRA-PCR that could detect both mutations in each molecular form. Three primers i.e., Sub_kdF (universal), SubA_kdR (specific to Form A) and SubB_kdR (specific to Form B), were designed for PIRA-PCR. The universal primer Sub_kdF is a modification of primer SubF designed by Singh et al. [19] wherein a single mismatch (T>A) is incorporated on the third base from the 3´ end of the primer to create restriction site *Apo*I/*Xap*I (5′-R|AATTY-3´) in the PCR amplicon in case of presence of Phe codon (TTT or TTC) at L1014 residue. This primer a tail of 20 bp was introduced at the 5´ end to increase the size of the PCR-RFLP product for better resolution on the agarose gel. Two reverse primers SubA_kdR and SubB_kdR were designed based on sequences of molecular forms A and B, respectively. The primer SubA_kdR is identical to SubR [19] and has one intentional mismatch introduced to eliminate an *Apo*I/*Xap*I restriction site already present in the sequence (Table 1). The expected sizes of PCR amplicon for species for Form A and B are 122 bp and 114 bp respectively. The sizes of cleaved fragments upon restriction digestion (in the presence of the L1014F allele) are 74 and 44 bp in Form A, and 66 and 44 bp in Form B, excluding 4 bp single-strand overhang in each fragment.

**Fig 3:**
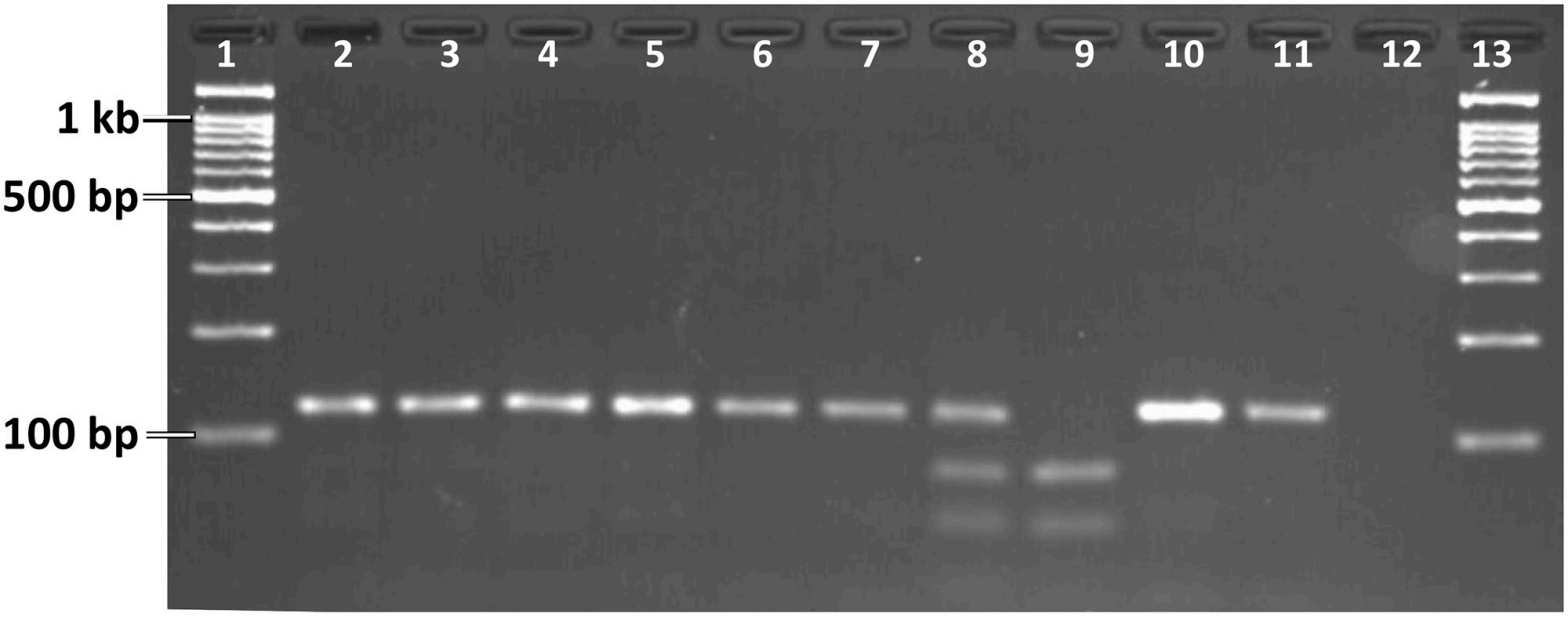
PIRA-PCR assay for *kdr* genotyping: Gel photograph showing undigested PCR products amplified from individuals belonging to Form A (lanes 2–4) and B (lanes 5–6), and *Apo*I-digested PCR products from individuals belonging to Form A (lanes 7–9) and B (lanes 10–12). Lane 7: LL, lane 8: LF, lane 9: FF; lane 10–11: LL; Lane 12: negative control without DNA; Lanes 1 & 13: 100 bp ladder.

The PCR amplification for the PIRA-PCR was carried out in a reaction mixture of 20 μl using GoTaq Green Master Mix 2X (Promega) and primers Sub_kdrF, SubA_kdr and SubB_kdr (0.25 μM each). The PCR conditions were: Initial denaturation at 95°C for 3 min followed by 35 cycles each with 95°C for 30 sec, 47°C for 30 sec, 72°C for 30 sec and a final extension at 72°C for 7 min. Five μl of PCR product was subjected to restriction digestion in a reaction mixture of 20 μl containing 2X CutSmart Buffer and 5 units of *Apo*I restriction enzyme (New England Biolabs Inc.). The reaction mixture was incubated at 50°C for 4 hours or overnight at room temperature. The product was electrophoresed on 2.5% agarose gel and visualized under UV (Fig 3).

### Statistical analysis

Pearson chi-square test was performed to compare differences in percent mortalities observed during bioassay using Microsoft Office-Excel. To establish the association of the *kdr* allele with insecticide resistance phenotype, the odd ratio (OR) and Fisher exact test were used.

## Results

### Insecticide susceptibility

**Fig 4:**
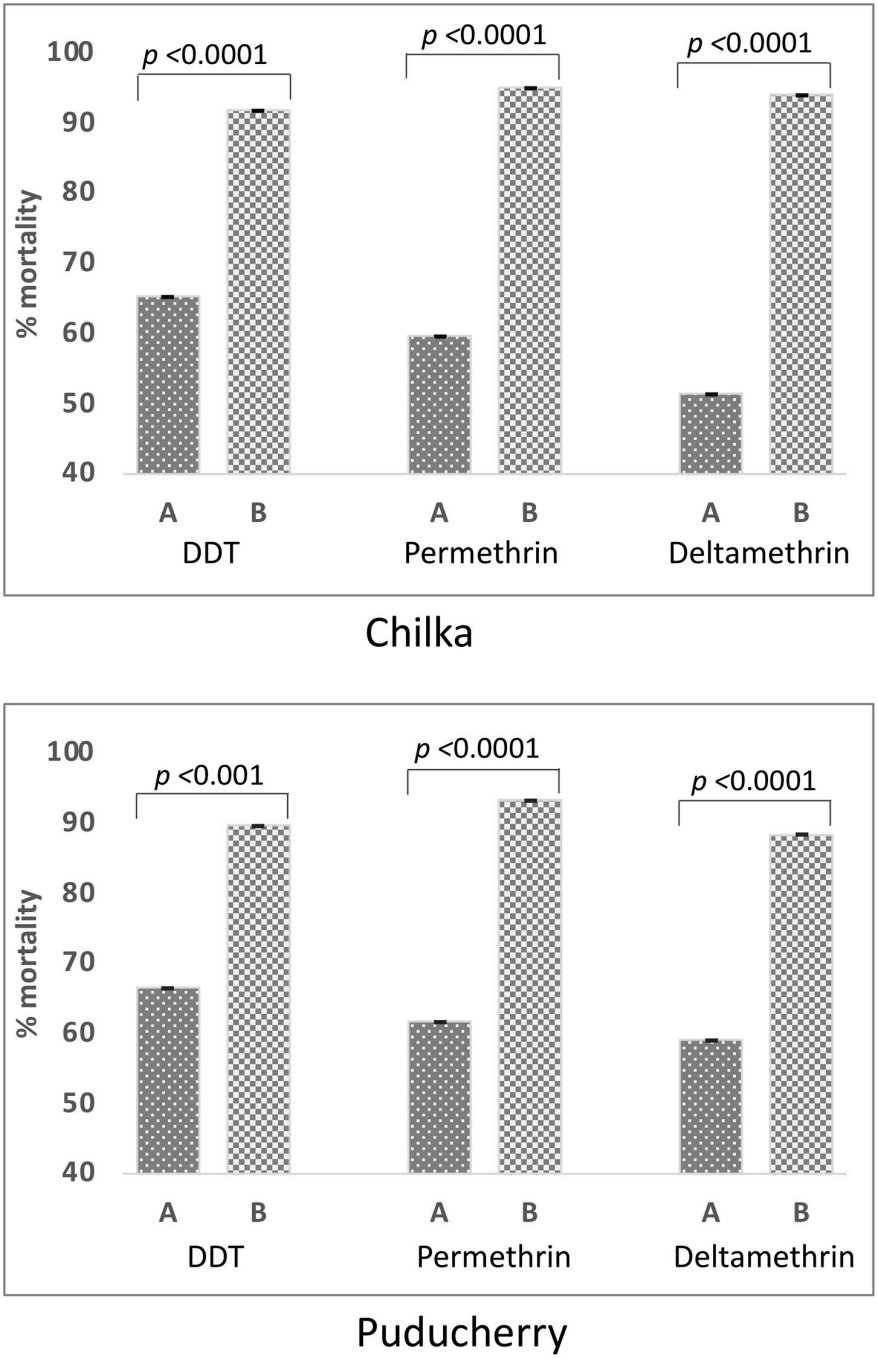
Bar chart showing relative corrected percent mortalities of two molecular forms of *An*. *subpictus* in two sympatric populations following standard WHO insecticide susceptibility tests against diagnostic doses of the insecticides. The *p*-value of the test of significance (chi-squared) between percent mortalities in Form A and B is shown on the top of bars.

The corrected percent mortalities in mosquitoes after 1-hour of exposure to diagnostic doses of insecticides along with the 95% confidence interval have been shown in Table 2. The molecular form A was resistant to all three insecticides tested and Form B was categorized under ‘possibly resistant’. Significantly higher mortalities in molecular form B from Chilka against DDT, permethrin and deltamethrin (91.9, 95.3 and 94.1, respectively) were observed as compared to Form A (65.5, 59.8, and 51.5 respectively) (Fig 4, panel A). Similarly, significantly higher mortalities in molecular form B against DDT, permethrin and deltamethrin (89.7, 93.4 and 88.5 respectively) were observed in bioassays as compared to Form A (66.7, 61.8 and 59.3, respectively) in Puducherry population too. (Fig 4, panel B). The Nuh population which is allopatric for Form A exhibited high resistance to all three insecticides exhibiting 20.4, 50.4 and 53.4 percent mortalities against DDT, permethrin and deltamethrin, respectively.

**Table 2:** Results of adult insecticide susceptibility tests carried out on two molecular forms of *An*. *subpictus*.

Geographical area	Collection date	Mol. Form	DDT 4%		Permethrin 0.75%		Deltamethrin 0.05%	
			N	% Corrected mortality* (95% CI)	N	% Corrected mortality* (95% CI)	N	% Corrected mortality* (95% CI)
Chilka	January 2021	A	111	63.96 (63.12–64.81)	102	60.78 (59.85–61.72)	106	50.94 (50.02–51.87)
	August 2019	A	66	66.67 (65.27–68.07)	68	58.82 (57.40–60.24)	66	51.52 (50.03–53.00)
	February 2018	A	59	66.10 (64.53–67.67)	69	59.42 (58.03–60.82)	79	51.90 (50.66–53.14)
	September 2018	A	45	66.67 (64.61–68.72)	67	59.70 (58.27–61.14)	110	51.82 (50.93–52.71)
	**Pooled**	**A**	**281**	**65.48 (65.15–65.81)**	**306**	**59.80 (59.49–60.12)**	**361**	**51.52 (51.25–51.79)**
	January 2021	B	109	92.66 (92.19–93.13)	98	97.96 (97.68–98.24)	94	94.68 (94.21–95.15)
	August 2019	B	85	91.76 (91.13–92.40)	116	94.83 (94.45–95.20)	116	93.97 (93.56–94.37)
	February 2018	B	37	91.89 (90.45–93.34)	71	94.37 (93.73–95.00)	74	94.59 (94.00–95.19)
	September 2018	B	40	90.00 (88.53–91.47)	53	92.45 (91.48–93.43)	72	93.06 (92.36–93.75)
	**Pooled**	**B**	**271**	**91.88 (91.68–92.08)**	**338**	**95.27 (95.14–95.39)**	**356**	**94.10 (93.97–94.23)**
Puducherry	February 2020	A	39	66.67 (64.30–69.04)	36	61.11 (58.46–63.77)	39	58.97 (56.50–61.45)
	November 2019	A	42	66.67 (64.47–68.87)	40	62.50 (60.13–64.87)	42	59.52 (57.23–61.81)
	**Pooled**	**A**	**81**	**66.67 (65.53–67.81)**	**76**	**61.84 (60.59–63.09)**	**81**	**59.26 (58.07–60.45)**
	February 2020	B	38	89.47 (87.89–91.06)	36	94.44 (93.20–95.69)	38	89.47 (87.89–91.06)
	November 2019	B	40	90.00 (88.53–91.47)	40	92.50 (91.21–93.79)	40	87.50 (85.88–89.12)
	**Pooled**	**B**	**78**	**89.74 (88.98–90.51)**	**76**	**93.42 (92.78–94.06)**	**78**	**88.46 (87.66–89.26)**
Nuh	September 2021	A	120	20.83 (20.17–21.5)	120	50.83 (50.02–51.65)	120	53.33 (52.52–54.15)
	October 2020	A	120	20.00 (19.35–20.65)	120	50.00 (49.18–50.82)	127	53.54 (52.77–54.31)
	**Pooled**	**A**	**240**	**20.42 (20.09–20.75)**	**240**	**50.42 (50.01–50.82)**	**247**	**53.44 (53.05–53.84)**

* Mortalities after one hour of exposure to insecticides followed by 24-hour recovery. Abbreviation used: N = number of mosquitoes exposed; CI = confidence interval

### Molecular characterization of partial voltage-gated sodium channel

**Fig 5:**
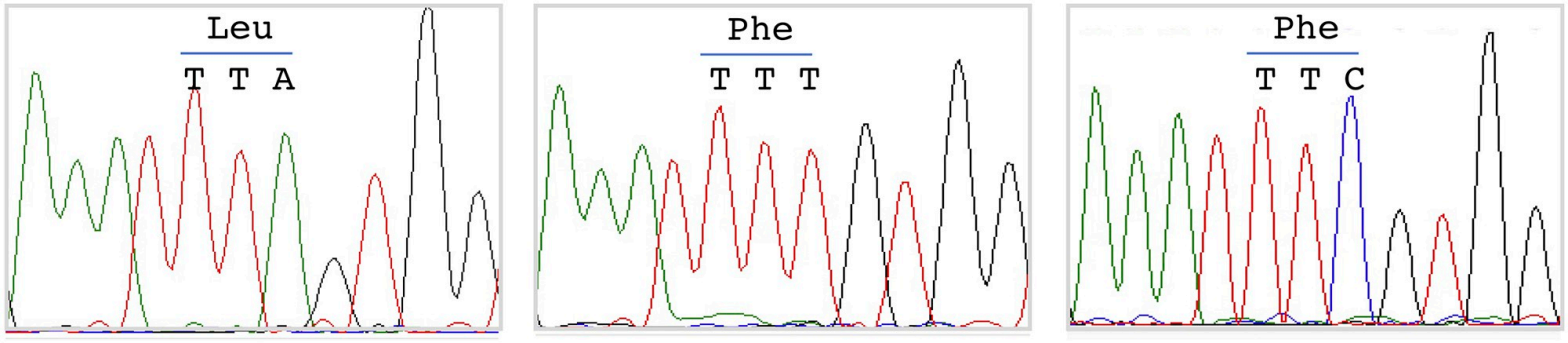
Snapshot of DNA sequence chromatograms showing three L1014 codons present in the VGSC of *An*. *subpictus* Form A.

A total of 70 representative samples of molecular form A and 42 samples of Form B were sequenced for the partial VGSC. Numbers of samples sequenced among the dead and alive mosquitoes following insecticide bioassays from Chilka and Puducherry have been provided in S1 Table. Two alternative transversions were recorded on the third base of L1014 codon (TTA), i.e., A>T and A>C, in molecular Form A, both leading to the same amino acid substitution, i.e., Leu→Phe (Fig 5). However, no nonsynonymous mutation was recorded in molecular Form B. The number of samples sequenced and their *kdr* genotypes are shown in Table 3.

**Table 3:** L1014-genotyping through DNA sequencing.

Location (Molecular form)	Codon at residue L1014 (amino acid)						
	TTA/TTA	TTA/TTT	TTA/ TTC	TTT/TTC	TTT/TTT	TTC/TTC	Total
	(L/L)	(L/F)	(L/F)	(F/F)	(F/F)	(F/F)	
Puducherry (A)	4	7	1	4	1	3	20
Chilka (A)	9	12	2	17	5	5	50
Puducherry (B)	10	0	0	0	0	0	10
Chilka (B)	32	0	0	0	0	0	32
**Total (A)**	**13**	**19**	**3**	**21**	**6**	**8**	**70**
**Total (B)**	**42**	**0**	**0**	**0**	**0**	**0**	**42**

### Frequency of the L1014F-*kdr* in molecular forms

The frequency of L1014F-*kdr* mutation in different populations as determined through PIRA-PCR has been shown in Table 4. The frequency of L1014F mutation in Form A ranged between 40% and 50% with the highest frequency recorded in the mainland population of Nuh (50%), followed by coastal populations of Puducherry (45%) and Chilka (40%). No *kdr* mutation was detected in Form B.

**Table 4:** L1014-*kdr* genotyping through PIRA-PCR.

	L1014-genotype				*kdr-*allele frequency	
	L/L	L/F	F/F	Total	L	F
Chilka (Form A)	40	38	21	99	0.596	0.404
Chilka (Form B)	26	0	0	26	1.000	0.000
Puducherry (Form A)	15	13	11	39	0.551	0.449
Puducherry (Form B)	23	0	0	23	1.000	0.000
Nuh (Form A)	36	70	38	144	0.493	0.507
**Total (A)**	91	121	70	282		
**Total (B)**	49	0	0	49		

### Association of *kdr* mutation with insecticide resistance phenotype

**Fig 6:**
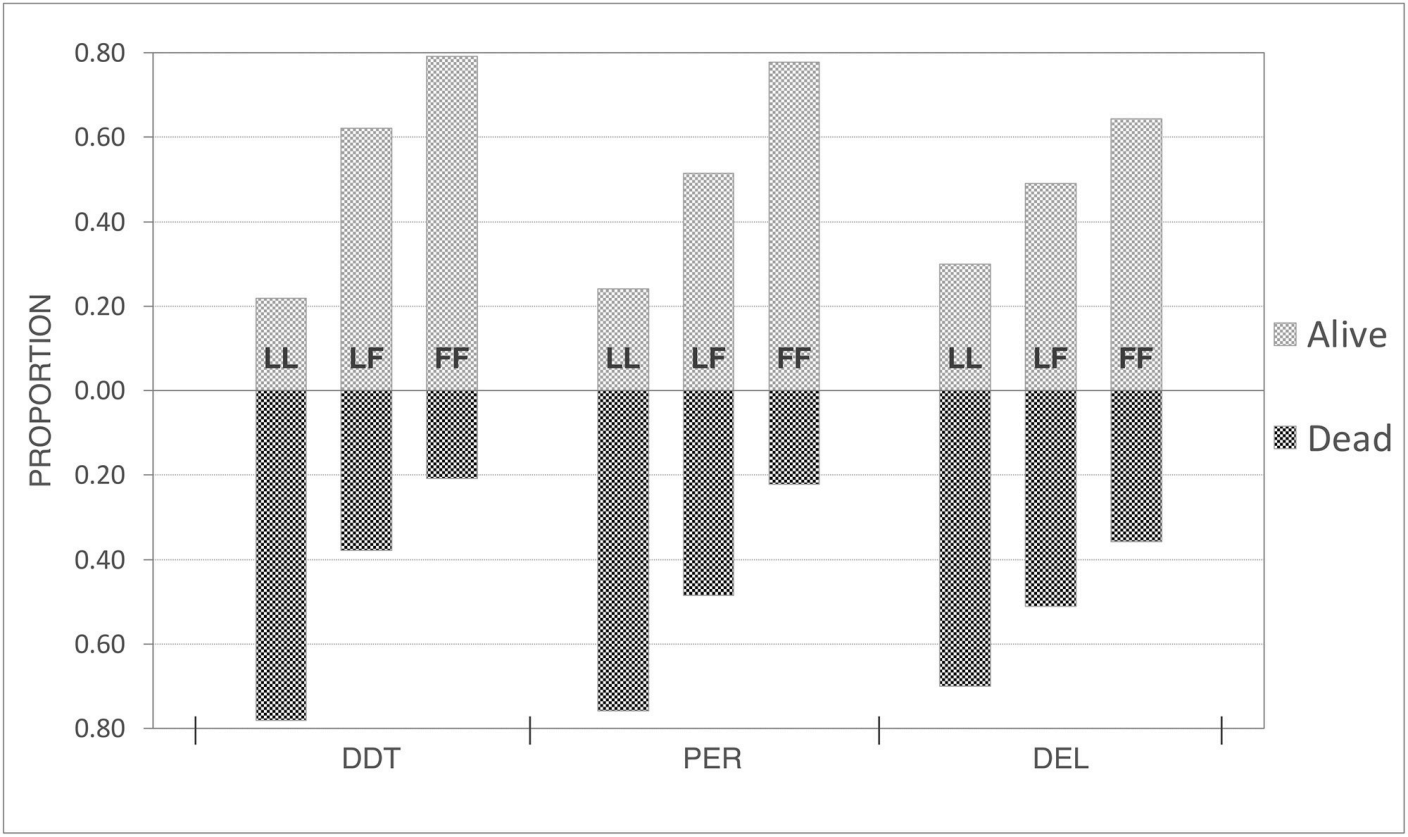
Bar chart showing the proportion of individuals with L1014 genotypes LL, LF and FF among dead and alive mosquitoes (Y-axis) following WHO’s standard insecticide susceptibility test with DDT 4%, permethrin 0.75% (PER) and deltamethrin 0.05% (DEL).

The proportions of L1014 and 1014F alleles in the dead and alive mosquitoes following WHO’s standard insecticide susceptibility test against DDT, deltamethrin and permethrin have been shown in Table 5 and Fig 6. The proportions of 1014F genotype were significantly higher in resistant mosquitoes as compared to susceptible counterparts following bioassay with DDT (OR: 4.4; 95% CI: 2.36–8.19; *p*<0.0001), permethrin (OR: 3.42, 95% CI: 1.79–6.57; *p*<0.001) and deltamethrin (OR: 2.13, 95% CI: 1.23–3.67; *p*<0.01).

**Table 5:** L1014-*kdr* genotype and resistance phenotype relationship among *An*. *subpictus* Form A.

Insecticide paper	Dead/alive	L1014-genotype				No. of alleles		Odd Ratio (95% CI)	*p* value (Fisher exact test)
		L/L	L/F	F/F	Total	L	F		
**DDT 4%**	Dead	25	14	5	44	64	24	4.40 (2.36–8.19)	< 0.0001
	Alive	7	23	19	49	37	61		
**Permethrin 0.75%**	Dead	22	17	4	43	61	25	3.42 (1.79–6.57)	< 0.001
	Alive	7	18	14	39	32	46		
**Deltamethrin 0.05%**	Dead	21	25	10	56	67	45	2.13 (1.23–3.67)	< 0.01
	Alive	9	24	18	51	42	60		

Abbreviations used: CI = confidence interval

## Discussion

The importance of recognition and identification of sibling species has been realized for decision-making process in vector control programme owing to differences in biological characteristics, such as host preferences, breeding behaviour, malaria vectorial competence, resting behaviour (outdoor/indoor) and responses to the insecticides. Significant differences have been reported in host preferences [20], role in malaria transmission [21], malaria sporogonic success (vectorial competence) [22–24] and insecticide resistance [25] among members of species complexes. Evidence suggests that members of the Subpictus complex exhibit differences in breeding preferences [8, 9, 17] and possibly in vectorial competence [6]. This study reports contrasting differences in insecticide resistance among two molecular forms of the Subpictus Complex and the occurrence of *kdr* mutations that confer resistance against DDT and pyrethroids. These two molecular forms are highly diverged to the extent that Form B is phylogenetically closer to the sundaicus complex; thus various authors [10, 12–13] have considered Form B as a member of the sundaicus complex. Therefore, it is important to identify the correct biological species for the effective management of vectors.

Not much information is available about insecticide resistance in Indian *An*. *subpictus* populations [2]. In Sri Lanka, Surendran et al. [26] have reported differential susceptibility of sibling species where species B was susceptible to DDT, malathion, deltamethrin and λ-Cyhalothrin. In another study in Sri Lanka, *An*. *sundaicus* s.l., i.e., species B of the *An*. *subpictus*, collected in Kilinochchi were completely susceptible to 0.05% deltamethrin and 5% malathion and resistant to 4% DDT, whereas those from Jaffna were relatively susceptible to all three insecticides [13]. Such differences were attributed to low insecticide selection pressure in coastal areas where species B is predominant [26]. In our study, we showed significantly higher levels of resistance in Form A as compared to Form B in sympatric populations which may have experienced similar insecticide selection pressure. We also noted higher levels of resistance in Form A populations present in inland populations as compared to coastal populations. A study carried out in Pakistan has shown almost similar resistance levels in *An*. *subpictus s*.*l*. against DDT, deltamethrin and permethrin [27] which may be probably molecular Form A, assuming that Form B is present in coastal regions only. The distribution of L1014-F *kdr* in molecular Form A also varied in different populations which were found to be higher in Nuh (51%) followed by Puducherry and Chilka with 45% and 40%, respectively. In another study [19] in the mainland (Jamshedpur, India), a high frequency of L1014F kdr mutation (68.69%) was recorded in Form A. We did not record the presence of *kdr* mutation in Form B. There are two reports of the presence of L1014F kdr mutation in Sri Lanka, one reporting TTA>TTC substitution in Jaffna city [28], and another TTA>TTA substitution in North Central Province [29], both leading to L1014F *kdr* mutation. The sequence analysis of DNA sequences generated through these two studies and submitted to GenBank revealed that the mosquitoes with *kdr* mutation belong to molecular Form A based on intron sequence. In this study, we recorded two alternative *kdr* mutations in all three populations of Form A but not in Form B. Though both molecular forms of subpictus in the coastal region might have experienced identical insecticide selection pressure, insecticide resistance and two independent kdr mutations evolved only in Form A. The possible explanation for this is the widespread presence of Form A in the Indian subcontinent which has a higher chance of selection of new genetic changes/mutations as compared to Form B which is restricted to coastal areas only.

The identification of sibling species is challenging in field studies. The chromosomal method is difficult to carry out due to various reasons, mainly, due to the requirement of live adult female mosquitoes with semi-gravid conditions which constitute a small proportion of a population and a difficult procedure that required a highly skilled technician to read polytene bands. The chromosomal technique has never been utilized in field studies for sibling species identification after Suguna [8] and Suguna et al. [9] except by Abhayawardana et al. [30] where they genotyped inversion present on a single locus (X+a and Xa) only which is not sufficient for species identification. For the correct identification species inversion genotype present on two diagnostic loci (Xa/+a and Xb/+b) is essential. The molecular tools on the other hand are simple and can be applied to alive as well as dead mosquitoes of either sex. An alternative to the cytological method is the median count of the number of ridges present on egg float, which is a cumbersome process and requires a lengthy procedure to obtain F1 isofemale progeny. In India and Sri Lanka, molecular characterization of *An*. *subpictus* revealed the presence of only two molecular forms based on 28S and ITS2 sequences which have been described as molecular forms A and B by Sindhania et al. [10] which can be identified by either of the two PCR methods developed by Surendran et al. [12] and Sindhania et al. [10], both of which are based on ITS2 sequence. Though these two methods can accurately differentiate Form A and B but have shown cross-reactivity to *An*. *stephensi* and *An*. *vagus* respectively, which are often misidentified as *An*. *subpictus s*.*l*. in specimens with lost hairs/scales. Therefore, in this study, we designed a new method to differentiate two molecular forms of *An*. *subpictus*. This method can be used for the large-scale identification of molecular forms. The molecular tools developed for the identification of molecular forms can be used in the vector control programme for vector surveillance which will aid to understand the seasonal dynamics and geographical distribution of different molecular forms of *An*. *subpictus*. Earlier studies have indicated the prevalence of two molecular forms/species of *An*. *subpictus* varies in different seasons [6, 8, 10]. The molecular tool will also help in defining the differential role of sibling species in malaria transmission which is hampered due to the technical difficulties associated with the conventional methods of sibling species identification, i.e., the cytotaxonomy and egg morphology.

The identification of genetic markers associated with resistance was included in the priorities of the WHO Global Plan for Insecticide Resistance Management [31]. In this study, we identified two point mutations at L1014 residue, both leading to L1014F mutation in molecular Form A as reported by Singh et al. [19] but such a mutation was absent in Form B. The M918T mutation, also referred to as super-*kdr*, was absent in both forms. We developed a common PIRA-PCR assay for the identification of L1014F mutation raised from two alternative transversions in both molecular forms. PIRA-PCR is advantageous over allele-specific PCR (AS-PCR) because it is highly specific due to the high specificity of the recognition site of specific restriction enzyme, whereas primers designed based on a single SNP are prone to non-specific extension [32, 33].

In this study, we showed that L1014F mutation is associated with resistance against DDT and pyrethroids. We observed that this mutation conferred significant protection against all three insecticides tested, i.e., DDT, permethrin and deltamethrin. Similar results were also observed during functional validation of this mutation in CRISPR/Cas9 modified *An*. *gambiae* which showed resistance against DDT (>24-fold) and pyrethroids [34]. In this study, they also showed that the L1014F has critical combined effect on resistance with the overexpression of glutathione S-transferase epsilon 2 (GSTe2) [34] which is known to have DDT-dehydrochlorinase activity [35]. Thus monitoring of *kdr* mutation along with other mechanisms of metabolic resistance is important in the vector control programme for effective insecticide resistance management.

One limitation of this study is that the number of mosquitoes tested for insecticide bioassay in one of the two coastal areas (i.e., Chilka) is not adequate as per WHO norms. However, the susceptibility status of molecular Form A and B as determined in this area is at par with the results obtained in another coastal area, i.e., Puducherry, and serves as a supplementary result.

In conclusion, the current study provides information on the differential susceptibility of molecular forms of *An*. *subpictus* against DDT and pyrethroids in addition to the presence or absence of L1014F-*kdr* mutation in sympatric populations which may have experienced a similar degree of insecticide exposure. Such information has an important bearing in vector management, especially of species B which is considered to be an important malaria vector in India.

## Supporting information

S1 Table L1014 genotypes in *An*. *subpictus* Form A as determined through DNA sequencing. (PDF)
