# Supplementary material for: Molecular forms of the Indian Anopheles subpictus complex differ in their susceptibility to insecticides and the presence of knockdown resistance (kdr) mutations in the voltage-gated sodium channel
Source: PLoS One. 2023 Feb 2;18(2):e0280289. doi: 10.1371/journal.pone.0280289 (PMC9894496; doi:10.1371/journal.pone.0280289)
Supplement: S1 Table — (PDF) [file pone.0280289.s001.pdf]

Table S1: L1014 genotypes in *An. subpictus* Form A as determined through DNA sequencing

Location (Molecular Form)		Codon at residue L1014 (amino acid)						
		Samples Sequenced (n)	TTA/TTA (L/L)	TTA/TTT (L/F)	TTA/ TTC (L/F)	TTT/TTC (F/F)	TTT/TTT (F/F)	TTC/TTC (F/F)
Puducherry (Form A)	DDT (dead)	1	1	0	0	0	0	0
	DDT (alive)	5	0	2	0	1	1	1
	DEL (dead)	3	1	1	0	1	0	0
	DEL (alive)	4	0	2	0	1	0	1
	PER (dead)	2	1	0	0	0	0	1
	PER (alive)	5	1	2	1	1	0	0
	Total	20	4	7	1	4	1	3
Chilka (Form A)	DDT (dead)	5	2	1	0	2	0	0
	DDT (alive)	11	1	3	0	4	2	1
	DEL (dead)	7	2	1	0	2	1	1
	DEL (alive)	12	1	3	1	4	1	2
	PER (dead)	4	2	1	0	1	0	0
	PER (alive)	11	1	3	1	4	1	1
	Total	50	9	12	2	17	5	5
Puducherry (Form B)	DDT (dead)	3	3	0	0	0	0	0
	DDT (alive)	1	1	0	0	0	0	0
	DEL (dead)	2	2	0	0	0	0	0
	DEL (alive)	1	1	0	0	0	0	0
	PER (dead)	2	2	0	0	0	0	0
	PER (alive)	1	1	0	0	0	0	0
	Total	10	10	0	0	0	0	0
Chilka (Form B)	DDT (dead)	7	7	0	0	0	0	0
	DDT (alive)	3	3	0	0	0	0	0
	DEL (dead)	10	10	0	0	0	0	0
	DEL (alive)	3	3	0	0	0	0	0
	PER (dead)	6	6	0	0	0	0	0
	PER (alive)	3	3	0	0	0	0	0
	Total	32	32	0	0	0	0	0
**Grand total (A)**		**13**	**13**	**19**	**3**	**21**	**6**	**8**
**Grand total (B)**		**42**	**42**	**0**	**0**	**0**	**0**	**0**
